# The impact of expanded brucellosis surveillance in beef cattle on human brucellosis in Korea: an interrupted time-series analysis

**DOI:** 10.1186/s12879-019-3825-6

**Published:** 2019-02-28

**Authors:** Sukhyun Ryu, Ricardo J. Soares Magalhães, Byung Chul Chun

**Affiliations:** 1 Division of Infectious Disease Control, Gyeonggi Provincial Government, Suwon, Republic of Korea; 2 Department of Epidemiology and Health Informatics, Graduate School of Public Health, Korea University, Seoul, Republic of Korea; 3 WHO Collaborating Centre for Infectious Disease Epidemiology and Control, School of Public Health, Li Ka Shing Faculty of Medicine, The University of Hong Kong, Hong Kong, Special Administrative Region China; 4 UQ Spatial Epidemiology Laboratory, School of Veterinary Science, The University of Queensland, Gatton, Australia; 5 Children’s Health and Environment Program, Child Health Research Centre, The University of Queensland, Brisbane, Australia; 6 Department of Preventive Medicine, Korea University College of Medicine, 73 Inchon-ro, Seongbukgu, Seoul, 02841 Republic of Korea

**Keywords:** Brucellosis, Policy, Prevention, Surveillance, Korea

## Abstract

**Background:**

Korean surveillance program for bovine brucellosis was improved by extending it to beef slaughterhouses and by pre-movement testing of bulls on May 2005 (Intervention 1). The bovine brucellosis surveillance program was further extended to beef cattle farms with more than 10 heads of cattle on June 2006 (Intervention 2).

**Methods:**

To quantify the temporal relationship between bovine and human brucellosis, a time-series analysis was conducted using Korean national notification data reported between January 2004 and December 2014.

**Results:**

Our findings indicate that while during the pre-intervention phase (January 2004 to March 2005) there was no significant temporal relationship between the incidences of bovine and human brucellosis, significant temporal relationships were observed after Intervention 1 (June 2005 to June 2006, no lag, *β* = 0.57, *p* = 0.04), and Intervention 2 (July 2006 to June 2007, 1-month lag, *β* = 0.65, *p* = 0.03). Furthermore, significant changes in incidence in human were observed after Intervention 1 (*β* = − 0.17 per 10 million-people, *p* = 0.03) and Intervention 2 (*β* = − 0.19 per 10 million-people, *p* = 0.04).

**Conclusions:**

These findings indicated the changes of a nationwide comprehensive surveillance programme targeting all cattle is required for effective reduction in the human population.

**Electronic supplementary material:**

The online version of this article (10.1186/s12879-019-3825-6) contains supplementary material, which is available to authorized users.

## Background

Brucellosis still remains a zoonotic disease of public health importance even in developed countries, causing a substantial loss of productivity in livestock industries and chronic disease in humans [1, 2]. Brucella is a gram-negative bacterium that can be transmitted to humans through direct contact of mucous membranes or broken skin with tissues from infected animals, ingestion of infected animals’ products such as raw meat or inhalation of infectious aerosols [3–5]. In the Republic of Korea, human brucellosis was first identified in 2002 in a 41-year-old male livestock farmer following the ingestion of unpasteurized milk [6]. Previous studies in Korea demonstrated that human brucellosis infections occurred as a result of exposure to infected cattle, and the most common strain of *Brucella* sp. is *Brucella abortus*, which is typically found in cattle populations [7–9].

In Korea, bovine brucellosis was first detected in 1955 in imported cattle originating from the US [10]. Between 1964 and 2003, control and prevention measures of bovine brucellosis mainly focused on dairy herds using the milk ring test on bulk milk samples [11–13]. Control measures including rapid depopulation were undertaken upon detection of a bovine case [11]. However, as a result of the reported number of case of human and bovine brucellosis in 2002, the surveillance of bovine brucellosis was expanded in 2004 to cover beef cattle. The beef cattle surveillance included mandatory pre-movement serological testing targeting areas with a high prevalence of bovine cases [13]. The expansion of bovine surveillance was accompanied by a proportionate increase in the depopulation of affected bovine cases, but this did not reduce the incidence of human and bovine brucellosis. Between March and June 2005, routine slaughter-house surveillance and mandatory testing of bulls prior to trade were introduced, which was followed, in June 2006, by the introduction of a mandatory nationwide herd testing of all beef farms with 10 or more heads of cattle [13, 14]. Control measures including removal of infected products and disinfection of facilities have been conducted [15]. Thus, due to the expanded surveillance in 2006, all dairy herds were screened annually using the milk ring test for dairy cattle, and 97% of beef herds were tested by using the serum agglutination test to identify possible Brucella infections [16].

While it is well known that the most effective strategy to control human brucellosis is through investments in surveillance and control programs to reduce brucellosis in bovine populations [17, 18], to date, no study has quantified the effect of successive implementation of different surveillance and control strategies of bovine brucellosis on the incidence of human brucellosis over time.

In this study, we aimed to quantify changes in the relationship between incidence of brucellosis in cattle herd and humans for the period between January 2004 and December 2014.

## Methods

### Case definitions

Since 2000, probable cases of human brucellosis have been defined using the following clinical and epidemiological criteria in patients identified at primary care centers and hospitals: (i) experiencing fever, malaise, muscle ache, or sweating and (ii) having occupations that put them at risk of direct contact with potentially infected animals (including farm workers, veterinarians, and abattoir workers). Confirmed cases of human brucellosis were defined as those that meet the definition of a probable case, and also tested positive for *Brucella* sp. on blood culture or antibody tests [19]. In July 2007, a direct polymerase chain reaction (PCR) test or second antibody test to identify the increased levels of antibody were added to the confirmed case definition [20].

For bovine brucellosis, during the study period, confirmed case was defined as cattle within the group of positive result from milk ring test or with positive on Rose Bengal Plate test, and identification of antibodies to *Brucella* sp. by standard tube agglutination test or competitive enzyme-linked immunosorbent assay [21].

### Data for analysis

Since 2000, human brucellosis has been designated as a Korean National Notifiable Infectious Disease, whereby all cases including probable and confirmed cases must be reported to the Korea Centers for Disease Control and Prevention (KCDC) [12]. Monthly reported numbers of cases of brucellosis in humans from physicians and cattle herds from veterinarians were collected from the Database of National Notifiable Infectious Diseases in the KCDC and the Korean Animal Health Integrated System for 11 years between 2004 and 2014 [22, 23]. The incidence of human brucellosis was calculated based on the number of reported human cases in the total population of Korea; and the cattle herd incidence of bovine brucellosis was measured based on the reported infected cattle herd number in the total cattle herd number of Korea during the study period [24].

### Statistical analysis

Considering the progressively improved surveillance of bovine brucellosis and the changes in laboratory criteria for the case definition of confirmed human brucellosis, the study period was divided into four phases comprising three sets of interventions. Phase 1 consisted of region-specific surveillance of beef cattle between January 2004 and March 2005. Phase 2 (June 2005 to June 2006) was considered as the period starting after implementing Intervention 1 which bovine surveillance was extended to slaughter houses and bulls prior to nationwide trade. Phase 3 (July 2006 to June 2007) was the period starting after Intervention 2 which extended surveillance on nationwide beef farms. Phase 4 (July 2007 to December 2014) was the period starting after Intervention 3 which introduced PCR in the diagnostic criteria of confirmed human brucellosis cases.

To identify temporal relationship between the monthly cattle herd incidence of bovine and monthly incidence of human brucellosis (monthly time-series over 132 months), we carried out cross correlation analysis, which generates a series of correlation coefficients between two-time series by shifting the two series temporally over a range of consecutive time lags [25]. This analysis is useful for determining the time lag, and maximizes the power of correlation between the two time-series [26]. For these time series, to remove trend and seasonality, the data are first independently and identically distributed, and subsequently, de-trended by differencing the time series at the degree of one. To examine the differences between before and after implementation of nationwide mandatory surveillance of bovine brucellosis, segmented regression analysis of time-series data from monthly incidence of human brucellosis was used. The trend of the incidence time series (defined as the difference of slope between before and after the intervention) and level changes in incidence (defined as the difference at the time point of intervention between the observed value and predicted value from pre-intervention time trend) were measured by using the following regression model [27–29]:

$$ {Y}_t={\beta}_0+{\beta}_1\times {time}_t+{\beta}_2\times Intervention{1}_t+{\beta}_3\times time\ after\ Intervention{1}_t+{\beta}_4\times Intervention{2}_t+{\beta}_5\times time\ after\ Intervention{2}_t+{\beta}_6\times Intervention{3}_t+{\beta}_7\times time\ after\ Intervention{3}_t+{e}_t $$

where, *Y*_*t*_ is the independent outcome variable of human brucellosis incidence at time *t* (the unit of time is the number of months); *β*_0_ estimates the baseline level of outcome at the initiation of time series; *β*_1_ is the coefficient of *time*_*t*_; *β*_2_ estimates the level of change in monthly incidence, where Intervention 1 = 0 is before June 2005 and Intervention 1 = 1 is after May 2005; *β*_3_ estimates the average monthly trend in the post-Intervention 1 period, where time after Intervention 1 is a continuous variable representing the number of months after June 2005 and is coded as zero before March 2005; *β*_4_ estimates the change in level of monthly incidence where Intervention 2 = 0 is before July 2006 and Intervention 2 = 1 is after June 2006; *β*_5_ estimates the average monthly trend in the post-Intervention 2 period, where time after Intervention 2 is a continuous variable representing the number of months after May2006 and is coded as zero before June 2006; *β*_6_ estimates the level of change in monthly incidence where Intervention 3 = 0 is before July 2007 and Intervention 3 = 1 is after June 2007; *β*_7_ estimates the average monthly trend in the post-Intervention 3 period, where time after Intervention 3 is a continuous variable representing the number of months after June 2007 and is coded as zero before July 2007; and *e*_*t*_ is the random effect at *time*_*t*_. Durbin-Watson test and analysis of residual plots were performed to identify any autocorrelation in the univariate time series model; this was followed by the application of a generalized least squares first order moving-average process model. The *astsa* package for R version 3.2.4 (R Foundation for Statistical Computing, Vienna, Austria) was used for the time-series analysis [30].

## Results

### Cross correlation between human and bovine brucellosis

**Fig. 1:**
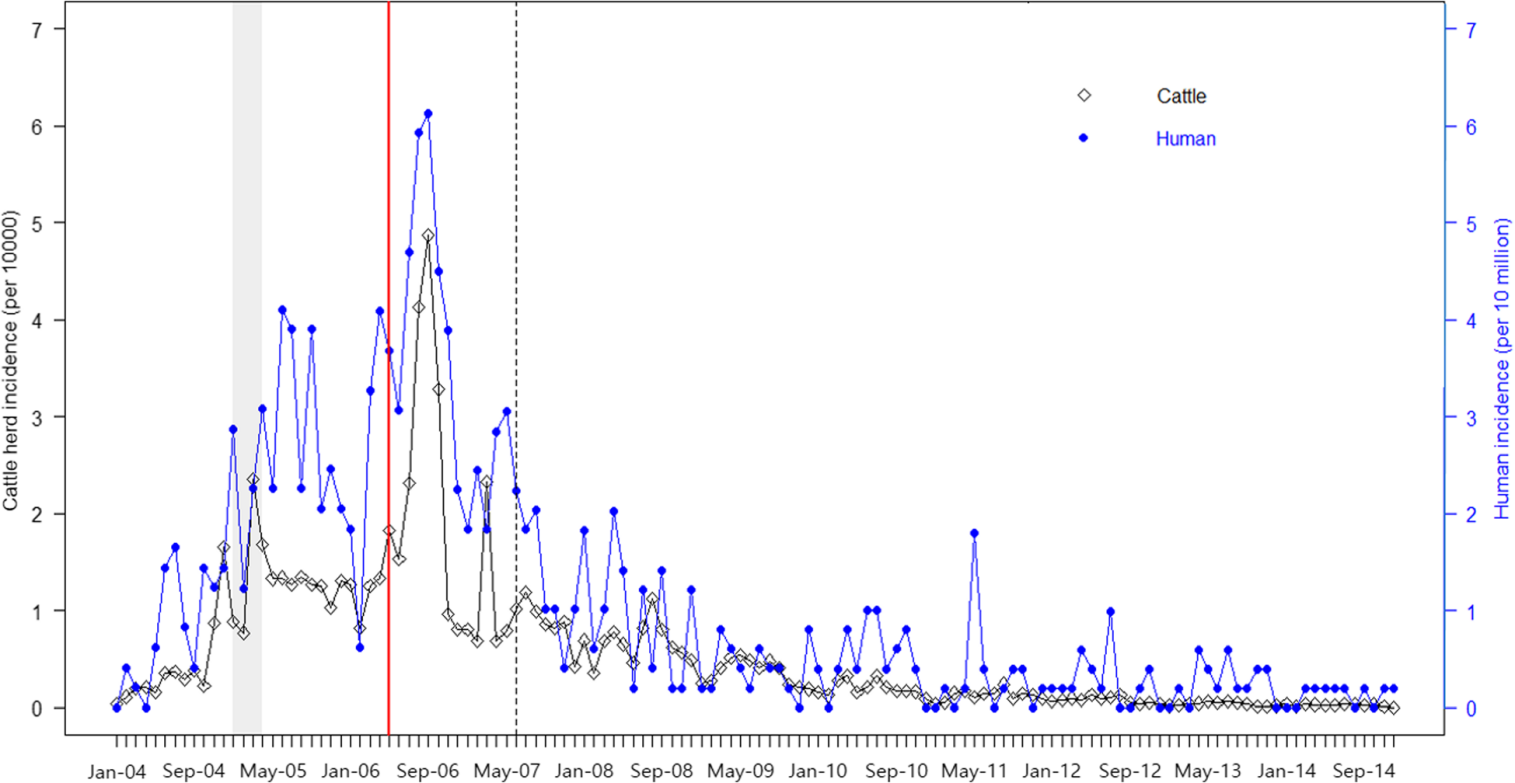
Monthly incidence of brucellosis in human and cattle herd between 2004 and 2014 in the Republic of Korea. The line with squares shows the incidence of brucellosis in cattle herd and the dotted line represents the incidence of human brucellosis. The gray vertical thick line represents Intervention 1 (March 2005 to June 2005), when expanded surveillance of slaughter houses of beef cattle and bulls prior to trade were implemented. The red vertical line depicts Intervention 2 (June 2006), when the surveillance of bovine brucellosis was extended to beef farms. The dotted vertical line represents Intervention 3 (July 2007), when the human case definition was changed to include the polymerase chain reaction assay and a second serological test

**Fig. 2:**
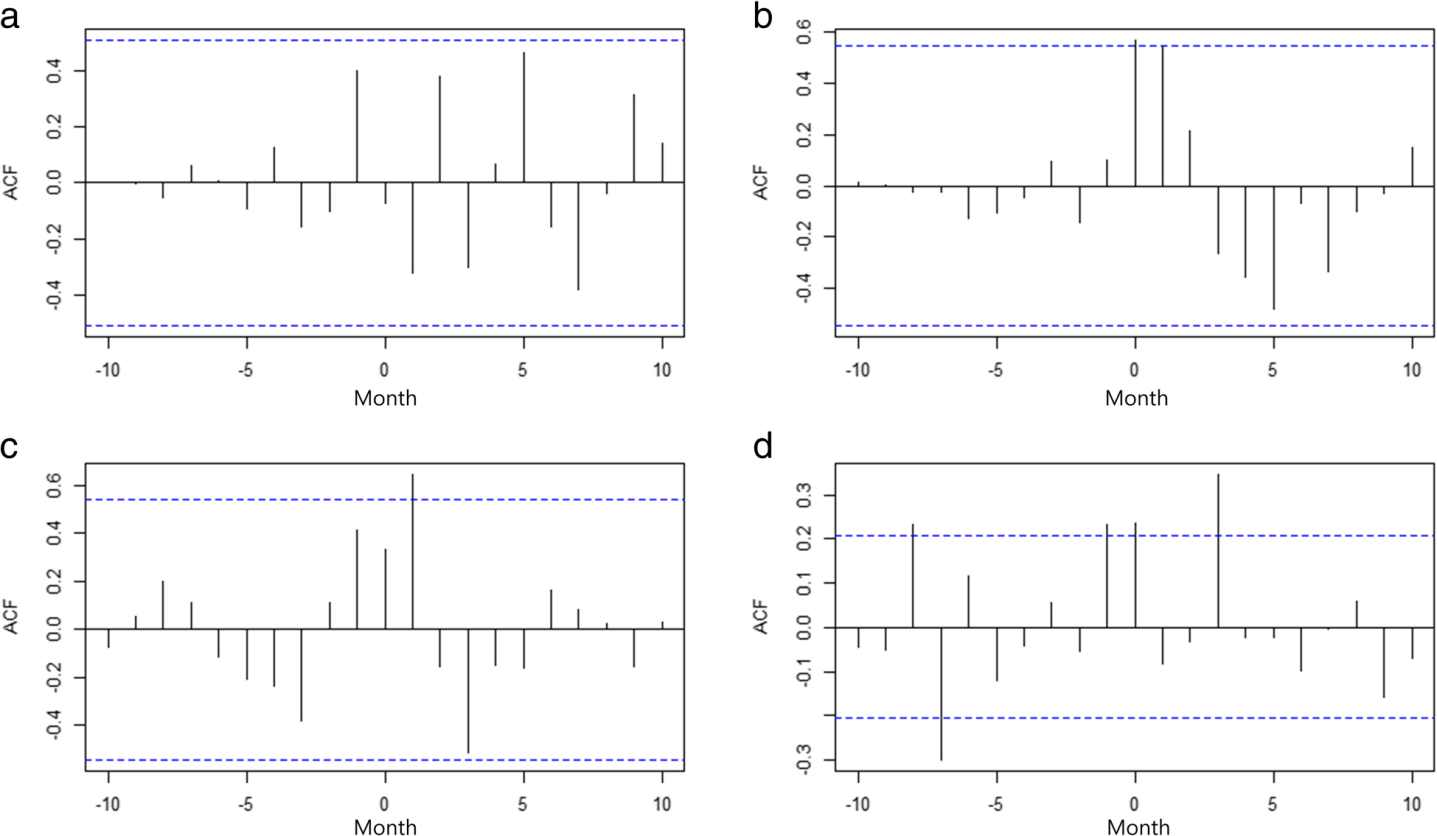
Results of cross-correlation function analysis for time series between the monthly incidence of brucellosis in human and cattle herd. This figure shows the cross-correlation function test for the residuals of the first-order-differenced data of brucellosis in human and cattle herd during the period of (**a**) Phase 1 (January 2004 to March 2005), (**b**) Phase 2 (June 2005 to June 2006), (**c**) Phase 3 (July 2006 to June 2007), and (**d**) Phase 4 (July 2007 to December 2014). The bars that cross the dashed horizontal lines (95% confidence interval) indicate considerable correlation between two-time series. **a** There was no significant cross-correlation during the initial stage of surveillance on beef cattle. **b** Significant cross-correlation without lag was observed after extending the surveillance to bulls prior to trade and to beef slaughter houses (*β* = 0.57, *p* = 0.04). **c** Significant positive cross-correlation was observed with a 1-month-lag after extending the surveillance to all beef farms with 10 or more heads (*β* = 0.65, *p* = 0.03). **d** Significant cross correlation with various month-lags is observed after adding the polymerase chain reaction diagnostic method on the definition of human case (a positive 3-month-lag: *β* = 0.35, *p* = 0.01; 0-month-lag: *β* = 0.24, *p* = 0.03; a negative 1-month-lag: *β* = 0.23, *p* = 0.03; negative 7-month-lag (*β* = − 0.30, *p* = 0.02; negative 8-month-lag: *β* = 0.23, *p* = 0.03). ^†^Autocorrelation function (ACF)

Between 2004 and 2014, the incidence of human and the cattle herd incidence of bovine brucellosis increased from January 2005 to July 2006 and decreased thereafter (Fig. 1). During Phase 1 (January 2004 to March 2005), there was no significant cross correlation between cattle herd incidence of bovine and the incidence of human brucellosis (Fig. 2a). However, during Phase 2 (June 2005 to June 2006), a significant temporal relationship (*β* = 0.57, *p* = 0.04) without lag was observed (Fig. 2b). During Phase 3 (July 2006 to June 2007), a significant positive relationship with a 1-month-lag (*β* = 0.65, *p* = 0.03) was found (Fig. 2c). During Phase 4 (July 2007 to December 2014), significant positive temporal relationships with a 3-month-lag (*β* = 0.35, *p* = 0.01), 0-month-lag (*β* = 0.24, *p* = 0.03), and negative temporal relationship with a 1-month lag (*β* = 0.23; *p* = 0.03), a 7-month lag (*β* = − 0.30, *p* = 0.02), and an 8-month lag (*β* = 0.23, *p* = 0.03) were identified (Fig. 2d).

### The impact of expanded surveillance of beef cattle on human brucellosis

There was an increase in the trend of incidence (0.14 cases per 10 million people; *p* <  0.01) during Phase 1 (Table. 1). During Phase 2, immediate increase in the incidence level (0.76 per 10 million people; *p* = 0.18) after Intervention 1 and decrease in the incidence trend compared with Phase 1 (− 0.17 per 10 million people; *p* = 0.03) were identified. During Phase 3, the incidence level was increased (1.94 per 10 million people; *p* <  0.01) immediately after Intervention 2 and the trend was decreased (− 0.19 per 10 million people; *p* = 0.04). During Phase 4, there was an immediate decrease in the incidence level (− 1.02 per 10 million people; *p* = 0.04) after Intervention 3 and increasing slope of incidence compared with Phase 3 was identified (0.20 per 10 million people; *p* <  0.01).

**Table 1:** Results of segmented regression analysis of human brucellosis by expanded surveillance of bovine brucellosis in beef cattle and adding diagnostic methods for the case definition of human brucellosis

Parameter	Value^a^	95% Confidence Interval	*p*-value
Pre-intervention phase			
Pre-intervention slope (secular trend, per month)	0.14	0.10 to 0.18	< 0.01
Intervention 1			
Change in level (immediate effect)	0.76	0.20 to 1.32	0.18
Change in slope (gradual effect per month)	−0.17	− 0.25 to − 0.09	0.03
Intervention 2			
Change in level	1.94	1.40 to 2.53	< 0.01
Change in slope	−0.19	− 0.28 to − 0.10	0.04
Intervention 3			
Change in level	−1.02	−1.51 to −0.53	0.04
Change in slope	0.20	0.14 to 0.26	< 0.01

^a^Incidence: per 10 million people

## Discussion

To date there were no published studies documenting this decay and the correlation between the intervention in bovines and the resulting decline in human incidence. Previous studies into human brucellosis in Korea demonstrated a significant linear correlation between yearly incidence of human and bovine brucellosis during the study period (2002–2009; and 2001–2011) [12, 31]. However, considering changes in surveillance and use of yearly incidence data with linear trend, interpretation of results of these previous studies may be limited. In this study, we analyzed the monthly incidence data of human and cattle herd brucellosis notifications, and decomposed the study period into several phases by taking into account successive implementation of enhanced cattle surveillance periods and addition of more sensitive diagnostic criteria of human brucellosis. Overall our study demonstrates that as the surveillance programme for Brucellosis in Korea expanded this was associated with a concomitant decrease in the incidence trend and level of reported human brucellosis infections. These findings have important implications on how brucellosis control could be designed and operationalized in other endemic countries by demonstrating that the efficacy of progressive expansion of a brucellosis surveillance and control programme in livestock can contribute to a stepwise decrease in human incidence.

In this study, we applied an analytical approach that included a test of cross-correlation and segmented regression analysis enabling an assessment of the strength of association between reported incidence of human and cattle herd incidence of bovine brucellosis and an evaluation of changes in the trend of the incidence of human brucellosis. Our results suggest that there was no significant temporal relationship between the incidence of human and the cattle herd incidence of bovine brucellosis during Phase 1, which can be partially explained by the fact that beef cattle surveillance during that period was primarily region-specific. This indicates that surveillance of bovine brucellosis that focused on dairy cattle and partial surveillance of beef cattle for Phase 1 had limited impact on the reduction of human brucellosis incidence. This is consistent with a previous article demonstrated that surveillance of zoonoses should cover both human and animal populations to increase the probability of early disease detection and for immediate effective prevention and control measures [32].

During Phase 2 (after Intervention 1, when beef cattle surveillance was extended to slaughterhouses and bulls), our results demonstrated a significant temporal correlation between the incidence of human and the cattle herd incidence of bovine brucellosis. This finding is consistent with that from a previous Korean study [33], which found that workers at slaughterhouses and markets of beef by-products were at risk for human brucellosis infection. During this period, a significant decreasing trend in the incidence of human brucellosis was found. This is likely due to the eradication program of bovine brucellosis infected bulls and the trading of brucellosis-negative beef cattle in livestock markets. During Phase 3 (after Intervention 2, where the surveillance extended to all beef farms), we observed a positive temporal correlation with a 1-month lag, indicating that the time series of human incidence is a month behind the incidence of bovine brucellosis. Moreover, an immediate positive level change and decreasing trend in the incidence of human brucellosis were found. It is likely that these results were affected by the proactive nationwide surveillance of beef farms. During Phase 4 (after Intervention 3, which included the addition of PCR in the human diagnostic criteria), several temporal correlations were found at various month lags. In addition, a negative level change and increasing trend compared with Phase 3 in the incidence of human brucellosis were found. Given that the occurrence of human brucellosis cases should follow that of bovine brucellosis, additional studies are required to understand the conditions that could have contributed to this finding.

During our study period, a number of studies reported that most cattle farmers in Korea were at high risk of exposure of infectious diseases because of inappropriate wear of protective glasses (> 90% in 2006, and 72% in 2013) and apron (91% in 2013) [34, 35]. Furthermore, the number of cattle herds in Korea remained stable between year 2004 (188,987) and year 2010 (176,021) [24]. These findings suggest that the risk of exposure among cattle farmers and the number of cattle herds is likely not to have had a significant impact on the incidence of brucellosis in human and cattle herd.

The findings of the present study need to be interpreted in light of some limitations. First, this was an ecological study that could not measure the causal association between bovine brucellosis prevalence and human brucellosis notifications. However, previous studies in Korea have shown that more proximal factors such as individual-level occupation is a major risk factor of human brucellosis [20]; other studies demonstrated that foodborne exposure to brucella has a role acquiring human brucellosis [4, 5]. Second, based on the case definition, we only considered individuals with human brucellosis-related symptoms, which have led to under-reporting of cases. Furthermore, we measured cattle herd incidence of bovine brucellosis based on the number of reported infected cattle herds which does not reflect the individual incidence level. Third, while interrupted time-series analysis has been regarded as a useful method for the evaluation of public health intervention at a population level [36], this method applied without a suitable comparison group and thereby does not allow the estimation of a causal association between brucellosis interventions and brucellosis incidence. Finally, it is possible that differences in the proportion of probable and confirmed cases of human brucellosis occurred over time after introduction of PCR assay to the case definition of confirmed cases; however, since the KCDC database provided the number of cases as a sum of probable and confirmed cases, it was not possible to ascertain the relative proportions for each of the case definition. Nevertheless, the proportion of probable cases was reported to be approximately 3.6%, and this database is often used as a proxy for measures of the incidence of human brucellosis in Korea [37].

## Conclusions

This study demonstrates that the incidence of human Brucellosis in Korea decreased as the surveillance programme of bovine brucellosis expanded. This finding indicates that a comprehensive surveillance programme targeting all cattle is required for effective brucellosis morbidity reduction in the human population.

## Additional file

Additional file 1: Monthly incidence of brucellosis in human and cattle herd in Korea. The supplementary file provides a dataset of monthly incidence of brucellosis in human and cattle heard in Korea used for assessing the temporal relationship between human and bovine brucellosis and measuring the effect of successive implementation of difference surveillance and control strategies of bovine brucellosis on the incidence of human brucellosis. (CSV 4 kb)

## Abbreviations

KCDC: Korea Centers for Disease Control and Prevention
PCR: Polymerase chain reaction
